# Endothelial cell talin1 is essential for embryonic angiogenesis

**DOI:** 10.1016/j.ydbio.2010.11.010

**Published:** 2011-01-15

**Authors:** Susan J. Monkley, Vassiliki Kostourou, Lorraine Spence, Brian Petrich, Stacey Coleman, Mark H. Ginsberg, Catrin A. Pritchard, David R. Critchley

**Affiliations:** a Department of Biochemistry, University of Leicester, Lancaster Road, Leicester, LE1 9HN, UK; b Vascular Adhesion Laboratory, BSRC Al. Fleming, Athens, Greece; c Department of Medicine, University of California, San Diego, La Jolla, CA 92093-0726, USA

**Keywords:** Angiogenesis, Cell adhesion, Endothelial cells, Talin1, Talin2

## Abstract

Using *Tln1*^*fl/fl*^*;CreER* mice, we show that tamoxifen-induced inactivation of the talin1 gene throughout the embryo produces an angiogenesis phenotype that is restricted to newly forming blood vessels. The phenotype has a rapid onset in early embryos, resulting in vessel defects by 48 h and death of the embryo within 72 h. Very similar vascular defects were obtained using a Tie2-Cre endothelial cell-specific *Tln1* knockout, a phenotype that was rescued by expression of a *Tln1* mini-gene in endothelial cells. We show that endothelial cells, unlike most other cell types, do not express talin2, which can compensate for loss of talin1, and demonstrate for the first time that endothelial cells in vivo lacking talin1 are unable to undergo the cell spreading and flattening required to form vessels.

## Research Highlights

► Endothelial cells express only talin1 and not the highly related talin2. ► Inactivation of the talin1 gene in the embryo results in a rapid onset angiogenesis defect. ► *Tln1* null endothelial cells in vivo are unable to form lumen-containing vessels. ► Talin1 and integrin-mediated adhesion are required from the earliest stages of angiogenesis.

## Introduction

Adhesion of animal cells to the extracellular matrix (ECM) is essential for the development of multicellular organisms and for the functional and structural integrity of tissues in the adult (Hynes, 2009). Cell-ECM interactions are mediated primarily by the integrin family of cell adhesion molecules, transmembrane receptors composed of an α- and β-subunit. Integrins possess a large extracellular domain that interacts with ECM proteins and a small intracellular domain that interacts with a wide variety of proteins inside the cell including signalling and actin-binding proteins (Legate et al., 2009; Moser et al., 2009). The adaptor protein talin is one of a number of proteins that couples the cytoplasmic tail of the β-integrin subunit to F-actin, a link that is required to transmit force from the actin cytoskeleton to the extracellular matrix (Critchley, 2009). However, in addition to its structural role, talin also functions as a regulator of integrin activity by binding to β-integrin tails in a two-step process that alters the conformation of the integrin heterodimer and increases its affinity for extracellular ligand (Anthis et al., 2009; Shattil et al., 2010; Wegener et al., 2007). The ability of talin to activate integrins allows it to regulate the assembly of multi-protein adhesion and signalling complexes (focal adhesions; FA) that are required for cell spreading, migration and contraction (Zhang et al., 2008).

Talin is a large (270 kDa) dimeric adaptor protein made up of an N-terminal head domain and a long flexible rod domain that can be dissociated from each other by calpain 2 cleavage (Critchley, 2009. The talin head comprises an atypical FERM domain (Elliott et al., 2010) that contains the major integrin-binding site (Anthis et al., 2009; Bouaouina et al., 2008; Wegener et al., 2007), as well as binding sites for signalling proteins such as the type 1ϒ isoform of PIP-kinase (Barsukov et al., 2003; Di Paolo et al., 2002; Ling et al., 2002) and also acidic phospholipids (Anthis et al., 2009; Goult et al., 2010). The talin rod is made up of a series of amphipathic helical bundles and contains a second integrin-binding site of as yet undetermined function (Gingras et al., 2009; Rodius et al., 2008), at least two actin-binding sites (Hemmings et al., 1996) and multiple binding sites for vinculin (Gingras et al., 2005) which itself can bind actin (Ziegler et al., 2006). The C-terminus of the rod contains the dimerisation domain and is required for maximal actin-binding (Gingras et al., 2008). The talin head and rod domains also interact intra-molecularly resulting in an autoinhibited form of the molecule that is thought to be cytoplasmic (Goksoy et al., 2008; Goult et al., 2009). Exactly how talin becomes activated is unclear, but there is strong evidence that this is regulated by a Rap1A/RIAM dependent signalling pathway (Han et al., 2006; Lee et al., 2009), and also by PIP2 (Goksoy et al., 2008).

There are two talin isoforms in vertebrates encoded by separate genes. Talin1 is required to maintain cell spreading, for cell migration and for FA formation (Kopp et al., 2010; Zhang et al., 2008). Talin2 was discovered following publication of the human genome sequence (Monkley et al., 2001) and appears to be encoded by the ancestral gene, with *Tln1* arising by gene duplication just prior to the emergence of vertebrates (Senetar and McCann, 2005). At the protein level, talin2 is 74% identical and 86% similar to talin1, and both talin isoforms are the same size and possess the same protein domains that are key to the function of talin1. At present, it is unclear why vertebrates express two such similar proteins. Evidence to date suggests that talin1 is expressed in all cells and tissues whereas talin2 is expressed in most but not all cell types (Debrand et al., 2009; Monkley et al., 2001; Senetar and McCann, 2005). Studies in cultured cells have shown that talin2 can functionally compensate for the loss of talin1 in cells that express both isoforms (Zhang et al., 2008), and talin2 can rescue the phenotype caused by loss of talin1 in cells where only this isoform is expressed (Kopp et al., 2010).

Gene knockout studies in mice have provided some insight into the roles of talin1 and talin2 at the organismal level. Constitutive knockout of talin1 results in developmental arrest at around gastrulation suggestive of an important role for this isoform in the early cell morphogenetic events that occur during embryonic development (Monkley et al., 2000). The severity and early onset of this phenotype has led to the assumption that talin2 is either not expressed in the early embryo, or performs a different role. Surprisingly, constitutive knockout of the *Tln2* gene either using a gene trap approach (Chen and Lo, 2005) or by deletion of the whole coding sequence produced no outward signs of any abnormalities during the first 18 months of life, and the mice are fertile (Debrand et al., unpublished data). Both talin isoforms are expressed in skeletal muscle, although talin2 is the predominant isoform, and co-localises with the β1D-integrin splice variant in myotendinous junctions (Conti et al., 2008). Consistent with this finding, knockout of *Tln2* produces a mild form of muscular dystrophy (although this was only apparent from histology), a phenotype that was significantly more severe than seen following muscle-specific deletion of *Tln1*. However, knockout of both genes led to severe defects in muscle development during embryogenesis that subsequently resulted in postnatal lethality (Conti et al., 2009). This suggests that in skeletal muscle, the two isoforms have overlapping and distinct functions. Similarly, tissue-specific inactivation of *Tln1* in heart also resulted in no apparent phenotype suggesting that compensation by talin2 may also occur in this tissue (R.S. Ross/A.-M. Manso, personal communication). In contrast, platelets express only talin1 and platelet-specific knockout of *Tln1* leads to spontaneous haemorrhaging and pathological bleeding (Nieswandt et al., 2007; Petrich et al., 2007).

The aim of this study was to establish whether any other cell types are dependent on talin1 alone, either due to lack of talin2 expression as in platelets, or due to functional differences between the two isoforms. To address this question, we temporally inactivated *Tln1* at different times during embryonic development using a tamoxifen-inducible CreER allele. The resulting loss of talin1 caused severe defects in angiogenesis without apparently affecting the development of other major tissues and organs. We show that this phenotype is due to loss of talin1 specifically from endothelial cells, which express only talin1 and not talin2, and that the defect in angiogenesis could be rescued by expression of an exogenous *Tln1* mini-gene.

## Material and methods

### Antibodies

PECAM1 antibody (MEC13.3) was from BD Pharmigen. Rat anti-endomucin (clone V7C7, Morgan et al., 1999) was a gift from Dr. Vestweber, Max Planck, Germany. Talin1 (97 H6) and talin2 (68E7) were generated in house (Debrand et al., 2009; Kopp et al., 2010). Anti-vinculin antibody (F9) was from Santa Cruz.

### Mouse strains and matings

All procedures on mice were performed in accordance with the UK Animals (Scientific Procedures) Act 1986. *Tln1* conditional mice (*Tln1*^fl^; MGI:3770513) have been described previously (Nieswandt et al., 2007; Petrich et al., 2007). Tie2-Cre mice (Kisanuki et al., 2001) were generously supplied by Dr. Yuri Kotelevtsev, Edinburgh. CreER (Hayashi and McMahon, 2002), and ROSA26lacZ (Soriano, 1999) mice were obtained from Jackson Labs and the CreER was activated in utero by one IP injection of the mother with 2 mg tamoxifen in corn oil. All mice were genotyped by PCR as described in Nieswandt et al. (2007) and Petrich et al. (2007).

*Tln1* transgenic mice were constructed using a vector consisting of a chicken actin promoter/CMV enhancer driving the expression of a loxP sequence-flanked EGFP cDNA followed by a mouse talin1 expression cassette (Fig. 6A). The talin1 expression cassette consisted of a mouse talin1 cDNA containing introns 2 and 3 and an amino-terminal hemagglutinin (HA) epitope. Founder mice were identified by PCR of genomic DNA using primers to GFP sequence (sense: 5'-GCACGACTTCTTCAAGTCCGCCATGCC-3', antisense: 5'-CCGTTCTTCTGCTTGTCGGCCATGATA-3') and observation of GFP fluorescence in various tissues with a fluorescence stereomicroscope (MVX10 Macroview, Olympus). A founder line selected for ubiquitous GFP expression and moderate, Cre-dependent HA-talin expression was crossed with *Tln1*^fl/fl^;*Tie2Cre* mice. Embryos were collected at the ages described based on the timing of the vaginal plug date, genotyped by PCR of yolk sac and digital photographs were obtained using stereomicroscopy.

### Whole mount embryo staining

Whole mount embryos were fixed in 2% paraformaldehyde (PFA), 0.1% glutaraldehyde for 10–20 min, washed and stained for β-galactosidase activity overnight at 37 °C in 2.5 mM X-gal, 5 mM potassium ferrocyanide, 5 mM potassium ferricyanide, 2 mM MgCl_2_, 2% sodium deoxycholate and 0.4% NP-40 in phosphate buffer. For PECAM1 immunostaining of whole embryos (with or without attached yolk sacs), embryos were fixed in 4% PFA for 2 h on ice then permeabilized in PBX (PBS, 0.1% Triton X-100) for 3 × 5 min at room temperature (RT). Embryos were dehydrated, then rehydrated through a methanol series and blocked in 10% normal goat serum (NGS)/PBX for 2 h RT with gentle agitation. They were then incubated in PECAM1 antibody (1:50) in 10% NGS/PBX overnight at 4 °C with gentle agitation followed by 5 × 1 h washes in PBX at RT. The washed embryos were then incubated in goat anti-rat Alexa 488 (1:400) in 10% NGS/PBX overnight in the dark at 4 °C with gentle agitation followed by 5 × 1 h washes in PBX at RT. Embryos were then post-fixed in 4% PFA in PBS for 10 min at RT and then washed in PBS. Stained embryos and yolk sacs were photographed either on a stereomicroscope with fluorescent illumination or imaged on a Leica TPS SP5 confocal microscope set up to detect Alexa-488 fluorophore. Deconvolution of the Z-series generated by confocal microscopy was performed using Huygens Essential Software and Imaris64 was used to generate both maximum intensity projections and 3D projection movies.

### Histology, immunoctyochemistry and electron microscopy

Embryos were fixed in 4%PFA followed by processing for wax embedding. Wax sections (5 μm) were de-waxed and, for histological analysis, were stained with hematoxylin and eosin, mounted and photographed. Sections to be used for immunohistochemistry were treated with 3% H_2_O_2_ in PBS to inactivate endogenous peroxidases followed by antigen retrieval using citric acid (pH 6) in a microwave for 12 min. The sections were then cooled and blocked in 10% NGS in PBX, then in 1:50 goat anti-mouse IgG antibody in PBS each for 1 h at RT. Sections were then incubated with endomucin antibody (1:10) in 10% NGS/PBS for 1 h at 4 °C, followed by 3 × 10 min washes in PBX, and then incubated with 1:200 biotin conjugated anti-rat secondary (BD Pharmingen) for 20 min at RT followed by washing for 3 × 10 min in PBX. Detection was performed by incubation in 1:500 streptavidin conjugated HRP (DAKO) in 10% NGS for 20 min at RT, followed by washing for 3 × 10 min in PBX and visualisation using Vector Labs DAB substrate kit. Sections were then lightly counterstained with haematoxylin, mounted and photographed (Leica DM5000B with DFC420C camera attachment).

Embryos to be analysed by EM were stained for β-gal as described above, fixed in 1% osmium tetroxide/PBS for 1 h, washed 3 × 20 min in distilled de-ionised water and processed through an ethanol series into 100% ethanol. Embryos were incubated in propylene oxide for 2 × 15 min then 50:50 propylene oxide/Spurr's resin (original formulation) for 2 h. The propylene oxide was allowed to slowly evaporate overnight and then replaced with fresh resin and incubated for 2 h (*x*2). Samples were placed into moulds and resin polymerised at 60 °C for 16 h. Embedded samples were remounted and sectioned transversely using a Reichert Ultracut S ultramicrotome. Thin sections of approximately 80 nm thickness were cut from each sample and collected onto copper mesh grids. The sections were counterstained with 2% Uranyl acetate for 20 min, followed by 4 min in Reynold's lead citrate. Samples were viewed on a JEOL 1220 TEM with an accelerating voltage of 80 kV. Images were captured using a Mageview III digital camera with Analysis software.

### Endothelial and fibroblast cell isolation and culture, staining and Western blotting

Mouse lung endothelial cells (MLEC) or embryonic endothelial cells were isolated and cultured according to Reynolds and Hodivala-Dilke (2006). Embryo fibroblasts were isolated and cultured according to Hüser et al. (2001). To activate CreER in cultured cells, 100 nM 4-hydroxy tamoxifen (4OHT) (or for controls the equivalent volume of ethanol) was added to the culture media at the time of plating and this was replaced 18–24 h later with fresh media. Western blotting was performed as previously described (Kopp et al., 2010). Cells plated onto fibronectin were stained with antibodies to talin1 (97 H6) or talin2 (68E7), PECAM1 and/or actin (Kopp et al., 2010).

## Results

### Inactivation of talin1 in mouse embryos leads to defects in angiogenesis

**Fig. 1:**
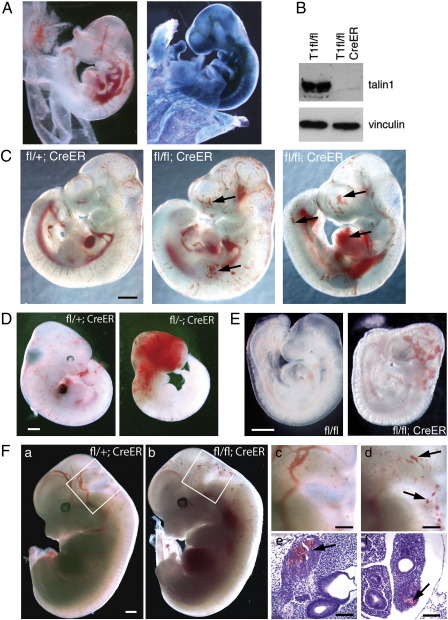
Tamoxifen-induced deletion of *Tln1* in mouse embryos results in widespread bleeding. (A) 10.5 dpc *Tln1*^fl/fl^;*CreER* embryo possessing the ROSA26 lacZ reporter exposed to tamoxifen in utero 48 h earlier (left panel) followed by staining with X-gal (right panel). (B) Mouse embryo fibroblasts of the genotypes shown were treated with 4OHT, and 96 h later cell lysates were blotted and probed with talin1 antibody 97 H6. Vinculin was used as a loading control. (C) Examples of 10.5 dpc embryos of genotypes shown exposed to tamoxifen in utero 48 h earlier at 8.5 dpc. Arrows indicate site of abnormal blood accumulation. (D) Examples of 11.5 dpc embryos of genotypes shown exposed to tamoxifen in utero 72 h earlier at 8.5 dpc. (E) 9.0 dpc embryos of genotypes shown exposed to tamoxifen in utero 48 h earlier at ~ 7.0 dpc. Bars = 0.5 cm. (F) 12.5 dpc embryos of genotypes shown exposed to tamoxifen in utero 48 h earlier. Boxed regions in (a) and (b) are magnified in (c) and (d), respectively. H&E stained sections of the embryo in (b) showing sites of bleeding (arrows) in ganglion VIII (e) and lung primordia (f). Bars = 0.5 cm (a–d) and 100 μm (e, f).

To investigate the role of talin1 in development of the post-gastrulation mouse embryo we crossed mice carrying the tamoxifen-inducible CreER transgene (Hayashi and McMahon, 2002) to conditional *Tln1* mice (Petrich et al., 2007; Nieswandt et al., 2007) to generate *Tln1*^fl/fl^;*CreER* embryos, and induced global *Tln1* inactivation in utero by maternal injection of tamoxifen. Littermates of other genotypes (e.g., *Tln1*^*fl/+*^;*CreER*) were used as controls. We also generated mice carrying the ROSA26 lacZ reporter (Soriano, 1999) to confirm that administration of tamoxifen to pregnant females efficiently activated CreER in embryos. As expected, tamoxifen-induced widespread expression of β-gal throughout the embryo (Fig. 1A). To demonstrate that Cre activation led to loss of talin1, we treated *Tln1*^fl/fl^;*CreER* mouse embryonic fibroblasts with 4-hydroxytamoxifen (4OHT); after 96 h very little talin1 was detectable compared to controls (Fig. 1B).

**Figure S1.**
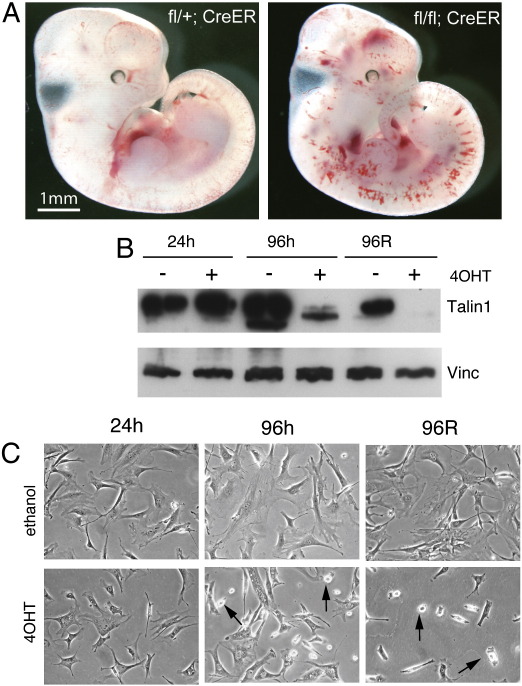
(A) 11.5 dpc embryos of genotypes shown, exposed to tamoxifen in utero 48 h earlier at ~ 9.5 dpc. (B) Western blot of protein lysates from *Tln1*^*fl/fl*^*;CreER* fibroblasts 24 or 96 h after they were treated with 4OHT (+) or ethanol (−). Cells at 96 h were also trypsinised, replated and lysates made 24 h after replating (96R). Blots were probed with 97 H6 talin1-specific antibody, with an anti-vinculin (Vinc) antibody serving as a loading control. (C) Morphology of *Tln1*^*fl/−*^*;CreER* fibroblasts treated either with 4OHT or ethanol vehicle. Cells were photographed at 24 and 96 h after addition of 4OHT/ethanol. Alternatively, cells were trypsinised at 96 h, replated and photographed a further 24 h later (96R). Arrows indicate cells that have failed to remain spread.

In the initial experiments, tamoxifen was injected into 8.5 dpc pregnant females and the embryos collected 24 or 48 h later. No phenotype was observed in *Tln1*^fl/fl^;*CreER* embryos collected at 24 h (*n* = 7; not shown). However, the 10.5 dpc *Tln1*^fl/fl^;*CreER* embryos collected 48 h after tamoxifen administration (referred to as 8.5/10.5 embryos) exhibited abnormal blood pooling at sites throughout the embryo (Fig. 1C). A similar phenotype was observed in 9.5/11.5 embryos (Fig. S1A). No such phenotype was observed in controls (Fig. 1C), or in embryos of any genotype where the mother was injected with corn oil vehicle instead of tamoxifen (not shown), indicating that the phenotype is associated specifically with the tamoxifen-induced loss of *Tln1*. If the 8.5/10.5 embryos were left for a further 24 h (total of 72 h post tamoxifen) to 11.5 dpc (8.5/11.5; *n* = 6) all embryos died, some exhibiting signs of severe haemorrhaging (Fig. 1D).

When tamoxifen was administered earlier, at approximately 7.0 dpc, and embryos examined 48 h later at 9.0 dpc (*n* = 8), the blood pools were severe and confined mainly to the head (Fig. 1E). The fact that maternal injection of tamoxifen at 7.0 dpc produced an early phenotype was unexpected since the placental connection between embryo and mother has not yet been established. We assume that the tamoxifen reached the embryo by diffusion across the yolk sac. In contrast, if tamoxifen is not administrated until 10.5 dpc and embryos collected 48 h later (10.5/12.5), the *Tln1*^fl/fl^;*CreER* embryos showed a very mild phenotype with only a few small bleeds visible on the surface of the head (Fig. 1F). Similar results were observed for 11.5/13.5 dpc and 12.5/14.5 dpc CreER embryos (not shown). Sectioning of 10.5/12.5 embryos showed that all the major structures and organs were present and appeared normal in both size and morphology, although localised haemorrhaging was observed in a range of tissues including lung, ganglion VII (Fig. 1F), head mesenchyme and neuroepithelium (not shown).

**Fig. 2:**
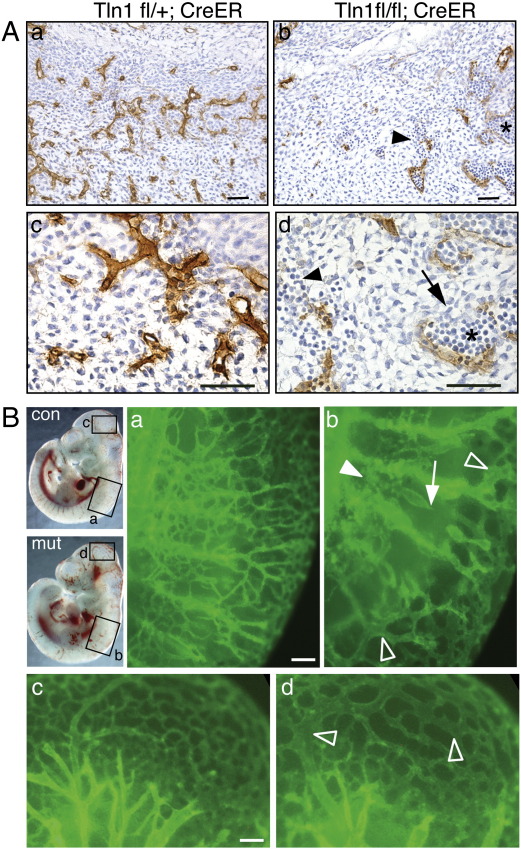
*Tln1* CreER embryos show defective blood vessel organisation. (A) 10.5 dpc embryos of genotypes shown were exposed in utero to tamoxifen at ~ 8.5 dpc and were then sectioned and stained with antibody to the endothelial-specific marker, endomucin (brown). Sections shown are of the lateral neural tube at the cervical region. The mutant vessels are often not completely enclosed (arrow) and packed with blood cells (*). In some areas blood cells can been seen outside the vessels (arrowhead). Bars = 50 μm. (B) 10.5 dpc *Tln1*^*fl/fl*^*;CreER* (mut) and *Tln1*^*fl/+*^*;CreER* (con) embryos exposed to tamoxifen in utero 48 h earlier were whole mount stained for PECAM1, then imaged on a fluorescent stereomicroscope; only boxed regions shown in left panels are shown (a–d). The mutant vessels (b, d) are dilated (open arrowhead), discontinuous (arrow) and endothelial cells are not organised into vessels (arrowhead). Bars = 100 μm.

To investigate the cause of the blood pooling/haemorrhaging in 8.5/10.5 dpc mutant embryos, they were sectioned and the blood vessels identified by staining for the endothelial cell marker endomucin (Morgan et al., 1999). The sections showed a reduction in the number of vessels, and those that were present were often irregularly shaped and sized, and often packed full with blood cells (Fig. 2A d; *), something never observed in control vessels (Fig. 2A a, c). There was also evidence of haemorrhaging (Fig. 2A d; arrowhead), and some vessels were not completely enclosed by endothelial cells (arrow). Whole mount staining of the 8.5/10.5 embryos for the vessel-specific protein PECAM1 (CD31) demonstrated a severely defective vascular structure throughout the mutant embryos. While the control 8.5/10.5 dpc embryo had a hierarchical network of regularly branching vessels (Fig. 2B a, c), the vessels of the mutant embryos were frequently dilated (Fig. 2B b, d; open arrowhead) and discontinuous, often ending abruptly in sac-like structures (arrow). They also displayed reduced branching and therefore decreased vascularisation of the surrounding tissues, and it appeared that some endothelial cells were not organised into vessels (Fig. 2B b; arrowhead). The above data point to an apparent defect in angiogenesis in *Tln1*^fl/fl^;*CreER* embryos exposed to tamoxifen.

Interestingly, the angiogenesis defect was the only phenotype observed in the 48 h following tamoxifen administration despite the fact that it would be expected to inactivate *Tln1* throughout the embryo. The other surprising observation is that the phenotype occurs so rapidly, i.e., within 48 h following exposure to tamoxifen. In cultured cells, at least 72–96 h are required after 4-hydroxy tamoxifen (4OHT) treatment before talin1 protein levels are low enough for defects in cell spreading to become apparent (Fig. S1B,C; Kopp et al., 2010). Trypsinisation and replating of cells to facilitate turnover of FA further reduces talin1 protein levels and increases the severity of the phenotype (Fig. S1C).

### Endothelial-specific inactivation of *Tln1* also results in angiogenesis defects

**Fig. 3:**
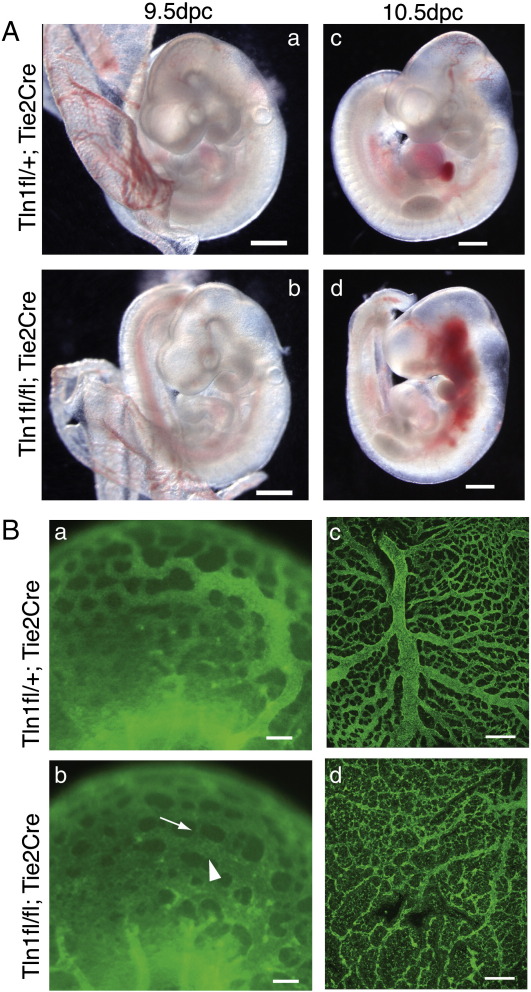
Endothelial cell-specific deletion of *Tln1* results in angiogenesis defects. (A) Examples of 9.5 dpc (a, b) and 10.5 dpc (c, d) embryos of genotypes shown. Bars = 0.5 mm. (B) PECAM1 whole mount stained 9.5 dpc embryo heads of genotypes shown (a, b) were imaged by fluorescent stereomicroscopy. While the vessels in the control (a) are uniform in size becoming narrower more distally, the vessels of the mutant (b) show large variations in diameter with both dilated (arrow) and narrow (arrowhead) vessels nearby one another. In the mutant there is also a variation in the size of the spaces between vessels. 9.5 dpc yolk sacs (c, d) of genotypes shown were also stained for PECAM1 and imaged by confocal microscopy; the maximum intensity projections are shown. Bar = 100 μm (a, b) and 200 μm (c,d).

Angiogenesis defects in embryos arise from deletion of a number of cell adhesion genes (Braren et al., 2006; Carlson et al., 2008; George et al., 1997; Proctor et al., 2005). In many cases this is due to defective endothelial cells, but in others it has been attributed to defects in the surrounding parenchymal cells or pericytes (Proctor et al., 2005; Silva et al., 2008). To assess the contribution of talin1 depletion from endothelial cells to the observed phenotype, we crossed *Tln1*^fl/fl^ mice to the endothelial cells-specific Cre line, Tie2-Cre (Kisanuki et al., 2001), to generate *Tln1*^fl/fl^;*Tie2-Cre* mice (referred to as Tie2-Cre mutants) in which *Tln1* is inactivated in endothelial cells and some blood cells. From a total of 71 animals (from 12 litters), no live *Tln1*^*fl/fl*^*;Tie2-Cre* animals were born, indicating that these mice die during embryogenesis. Embryos were collected from these matings, and while at 8.5 dpc the Tie2-Cre mutant embryos appeared indistinguishable from the control littermates, by 9.5 dpc approximately a third of the mutant embryos appeared slightly developmentally delayed and/or paler compared to the control embryos (Fig. 3A a, b). The 9.5 dpc Tie2-Cre embryos were also stained for PECAM1 and were imaged by fluorescent stereomicroscopy. The vessels in the heads of the Tie2-Cre mutants lacked the well-defined network of the controls, and instead, the vascular plexus had a flattened appearance, and the diameter of the vessels was not uniform unlike that of control vessels (Fig. 3B a,b; arrow and arrowhead). There was also variation in the size of the spaces between ‘vessels’ (Fig. 3B a,b). There was no evidence of any obvious heart defect in these mutant embryos either morphologically or histologically, and the hearts were beating at the time of dissection (not shown). By 10.5 dpc, the mutant embryos were dead or dying, often with evidence of severe haemorrhaging (Fig. 3A d). It was apparent at both 9.5 dpc and 10.5 dpc that the yolk sacs of the Tie2-Cre mutant embryos were much paler, thinner and more fragile than the controls (Figs. 3A a,b and 6B). PECAM1 staining of the 9.5 dpc yolk sacs highlighted defects in the vitelline vascular network of the mutants (Fig. 3B c, d) where only remnants of the major vessels were apparent. The vessels that were present appeared to have degenerated, and PECAM1 staining was weaker and abnormally distributed. This yolk sac phenotype was more severe than generally observed in the tamoxifen-treated CreER mutants.

### Talin1 is the only isoform expressed in endothelial cells

**Fig. 4:**
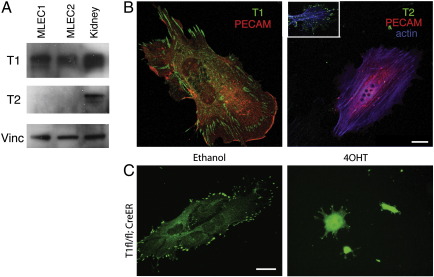
Talin1 is the only isoform expressed in endothelial cells and as such is necessary for cell spreading in vitro. (A) Western blots of lysates from two separate MLEC lines and embryonic kidney probed with talin1 (T1) and talin2 (T2) specific antibodies. Vinculin (Vinc) was used as a loading control. (B) Primary embryonic endothelial cells cultured on coverslips stained with antibodies to either talin1 (T1) or talin2 (T2) (green) along with antibodies to PECAM1 (red) and actin (blue). Talin2 was detected only in the PECAM negative non-endothelial cells (inset). (C) MLEC from *Tln1*^fl/fl^;*CreER* mice were treated with either 100 nM 4OHT or ethanol and stained 72 h later for talin1 (green). Bars = 20 μm.

Since Tie2-Cre-mediated knockout of *Tln1* in endothelial cells results in a similar angiogenesis defect to that seen following global inactivation of *Tln1* by CreER (compare Figs. 2B with 3B) it implied an endothelial defect in both cases so we conjectured that endothelial cells may only express talin1 and not the highly related talin2 isoform. To test this, we used talin1 and talin2 isoform-specific antibodies on isolated primary endothelial cells and confirmed that indeed talin1 was the only isoform detectable in mouse lung endothelial cells (MLEC) by Western blotting (Fig. 4A) and in embryonic endothelial cells by immunostaining (Fig. 4B). Talin1 was detected in FAs throughout the endothelial cell (Fig. 4B, left panel) whereas talin2 was detected only in FAs of the non-endothelial cells (Fig. 4B, right panel, inset). Similarly, talin2 was not expressed in primary human endothelial cells isolated from a range of vessels types including arterial, venous and microvessels and nor was it upregulated in talin1 knockdown endothelial cells (Kopp et al., 2010 and SJM unpublished data). The fact that endothelial cells only express talin1 whereas both talin1 and talin2 are expressed in embryonic fibroblasts (Kopp et al., 2010) would explain the endothelial cell-specific phenotype observed in the CreER mutant embryos exposed to tamoxifen. As talin1 is the only isoform expressed in endothelial cells, we used MLEC to look at the effect of *Tln1* knockout on the behaviour of these cells in culture. MLEC from *Tln1*^fl/fl^;*CreER* mice were treated with 4OHT (or vehicle control) and stained 72 h later for talin1. The 4OHT treated cells on glass coverslips were rounded and possessed no talin1-containing FAs, while cells treated with the vehicle control were flattened and spread, and possessed talin1-containing FAs (Fig. 4C).

### Endothelial cells lacking talin1 in vivo remain rounded and are unable to organise into vessels

**Fig. 5:**
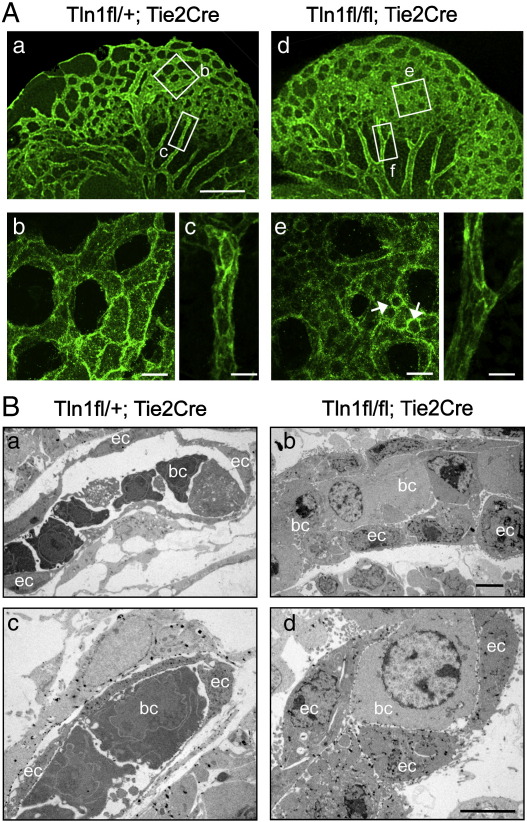
*Tln1* null endothelial cells are unable to spread and flatten in vivo. (A) Confocal maximum intensity projections of 9.5 dpc Tie2-Cre embryos whole mount stained for PECAM1 (genotypes shown). Higher power views of the boxed regions in (a) and (d) are shown in (b) and (c) and (e) and (f), respectively; Bars = 200 μm (a, d) and 20 μm (b, c, e, f). (B) Electron micrographs of ultrathin sections through the heads of X-gal stained 9.5 dpc embryos of genotypes shown. The endothelial cells (ec) and in some cases the blood cells (bc; which can also express *Tie2-Cre*) are identifiable due to the insoluble electron dense deposits of bromo-chloro-indolyl-galactoside product that appear as black spots within the cells. Bar = 5 μm.

To determine how loss of talin1 affects behaviour of endothelial cells in vivo, we looked closely at the morphology of the endothelial cells in newly formed/forming vessels in the heads of the 9.5 dpc *Tln1*^fl/fl^;*Tie2-Cre* embryos. Confocal imaging of PECAM1 stained embryos at high magnification allowed us to resolve the individual endothelial cells that make up the newly forming vessels (Fig. 5A b, e). In the control, the endothelial cells appeared large and flattened into tube-shaped vessels that enclosed a blood filled lumen (Fig. 5A b and Supplementary video 1). Endothelial cells in the mutant embryos were smaller and rounded (Fig. 5B e, arrows) and did not enclose a lumen but rather formed a sheet of cells (Supplementary video2). In contrast, the larger well-established vessels feeding the head region of the mutant embryos were composed of large flattened endothelial cells enclosing a lumen comparable to controls (Fig. 5B c, f).

Transmission electron microscopy (TEM) was used to confirm the above observations. In order to identify the endothelial cells in the EM sections, Tie2-Cre embryos were used that possessed the ROAS26-lacZ reporter (Soriano, 1999). These embryos were whole mount stained with X-gal prior to processing for EM. This allowed β-gal positive endothelial cells to be identified by their electron dense deposits of bromo-chloro-indolyl-galactoside (Nakajima et al., 2003). In control embryos, the vessels were composed of endothelial cells whose thin membrane extensions surrounded embryonic nucleated blood cells (Fig. 5B a, c). The endothelial cells comprising the vessels in the Tie2-Cre mutants were rounded, and there were no signs of the thin membrane protrusions. Again, as in the CreER mutant embryos, the blood cells were often tightly packed into vessels (Fig. 5B b, d). In summary, these results demonstrate for the first time that talin1 is required by endothelial cells in vivo to develop the spread morphology that is crucial for their organisation into vessels.

### The talin1 endothelial cell defect in vivo is rescued by expression of a talin1 mini-gene

**Fig. 6:**
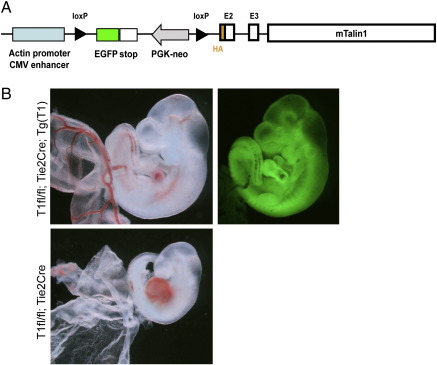
A *Tln1* mini-gene can rescue Tie2-Cre-mediated loss of *Tln1* in embryos. (A) Construct used to generate a transgenic line (*Tg*(*Gfp-Tln1*)) expressing GFP and Cre-inducible *Tln1* mini-gene. (B) 10.5 dpc embryos of genotypes shown were imaged on a stereomicroscope either under bright field (left) or GFP fluorescent illumination (right). Fluorescently imaged embryo shows widespread expression of GFP encoded by the transgene. The *Tln1*^fl/fl^;*Tie2-Cre* embryo at the bottom does not possess the GFP transgene and so no fluorescent image is shown.

A mouse line expressing a GFP cDNA and a *Tln1* mini-gene under the control of the actin promoter was generated by pro-nuclear injection. The construct was designed such that the *Tln1* mini-gene is only expressed upon Cre deletion of the floxed stop cassette containing the GFP cDNA (Fig. 6A). One line was chosen that expressed GFP in a wide range of tissues (not shown). We demonstrated that the transgene is also widely expressed in 9.5 dpc embryos as demonstrated by GFP fluorescence throughout the embryo (Fig. 6B). Crosses were established to generate Tie2-Cre mutant mice either with or without the GFP/*Tln1* transgene (*Tg(GFP-Tln1*). While the *Tln1*^fl/fl^;*Tie2-Cre* embryos do not survive to 10.5 dpc the additional presence of the GFP/*Tln1* transgene in embryos was able to rescue the angiogenesis defect and the *Tln1*^fl/fl^;*Tie2-Cre*;*Tg(GFP-Tln1)* embryos appeared normal with no evidence of vessel defects (Fig. 6B). The *Tln1* transgene was even able to rescue the Tie2-Cre mutant mice to birth, and 10 *Tln1*^fl/fl^;*Tie2-Cre*;*Tg(GFP-Tln1)* mice (compared to an expected 9; *p* = 0.15) were born that were viable and fertile and appeared indistinguishable from their littermates.

## Discussion

Tamoxifen-mediated global deletion of the *Tln1* gene in 8.5 dpc mouse embryos results in defects in blood vessel organisation 48 h later. The vascular defect is sufficiently severe that these embryos are not able to survive the subsequent 24 h (Fig. 1), presumably dying from lack of nutrient and oxygen supply. The surprising specificity and reproducibility of the vascular phenotype that resulted from deletion of *Tln1* throughout the embryo appears to be due to the fact that endothelial cells, unlike other embryonic cells (Kopp et al., 2010) and many adult tissues (Debrand et al., 2009; Monkley et al., 2001), only express talin1, and there is no talin2 to compensate for loss of talin1. The close similarity between the CreER and Tie2-Cre phenotypes suggests that the vascular phenotype in CreER embryos is due primarily to defects in endothelial cells that arise from loss of talin1. There is a possibility that CreER-mediated deletion of *Tln1* from other cells such as pericytes/vascular smooth muscle cells contributes to the phenotype, but this is unlikely as the vascular defects occur in newly forming vessels prior to the appearance of pericytes as determined by α-smooth muscle actin expression (not shown). In addition, primary aortic smooth muscle cells express both talin isoforms (Kopp, Monkley and Critchley, unpublished data), and therefore would not be expected to be affected by loss of talin1.

Numerous studies using both conditional and germline gene knockout of various integrin subunits have provided evidence that integrins play an important role in developmental angiogenesis, although it has been difficult to determine the contributions of specific integrins to this process due to complex phenotypes, and redundancy or compensation between certain integrin subunits (Hynes, 2007; Silva et al., 2008). For example α5- and αV-integrins have been shown to co-operate during vascular remodelling, but even when both these genes are inactivated in endothelial cells, the embryos are still able to form normal embryonic vasculature suggesting that additional α-integrin(s) can contribute to the process (van der Flier et al., 2010). Endothelial-specific deletion of β1-integrin produced the earliest and most severe defects of all integrins causing death of the embryos by 10.5 dpc (Carlson et al., 2008; Lei et al., 2008; Tanjore et al., 2008). The Tie2-Cre/β1-integrin phenotypes are very similar in nature and timing to the Tie2-Cre/talin1 phenotype reported here, in line with the idea that talins are critical for integrin-mediated adhesion. The absence of any vascular phenotype prior to 9.5 dpc in either talin1 or β1-integrin Tie2-Cre knockout embryos can be attributed to the delay between initial Tie2-Cre expression and the subsequent depletion of talin1 or β1-integrin protein. Evidence for this is provided here by the use of CreER to inactivate *Tln1* at 7.0 dpc in all endothelial cells (and precursors) prior to the time when they would first begin to express Tie2 (Kisanuki et al., 2001). This resulted in vascular abnormalities at 9.0 dpc (Fig. 1D) that were more severe than any observed with Tie2-Cre/talin1 (or Tie2-Cre/β1-integrin) confirming a role for talin1-mediated adhesion events from the earliest stages of angiogenesis. However we were unable to use this approach to investigate the role of talin1 in angiogenesis/vasculogenesis any earlier than 7.0 dpc because of the requirement for talin1 during pre-gastrulation morphogenesis (Monkley et al., 2000).

Inactivation of *Tln1* over a range of embryonic ages using the CreER/tamoxifen system has also allowed us to dissect more thoroughly the role of talin1 in older embryos that have already undergone significant vascularisation. In doing so, we observed that the later in development the tamoxifen was administered, the less severe and more localised the bleeding/vascular phenotype became. It appears that within the first 48 h following tamoxifen injection, *Tln1* inactivation in endothelial cells only affects newly forming vessels, while inactivation of *Tln1* in endothelial cells in established vessels had no apparent effect on vessel structure or integrity (Figs. 1F and 5A). This observation was surprising and we initially considered the possibility that talin1 may not required by endothelial cells once they become organised into vessels. However a more likely explanation is that endothelial cells in vessels have very stable cell–matrix junctions, and talin1 protein persists in these junctions long after deletion of the *Tln1* gene. In contrast, endothelial cells that are actively involved in angiogenesis and are thereby undergoing changes in cell shape and polarity will be continually remodelling their cell–matrix junctions, likely increasing the rate of talin1 protein turnover. As a result, their talin1 protein stocks will be depleted more rapidly, making these cells much more sensitive to inactivation of the *Tln1* gene. This conclusion is supported by observations on *Tln1* knockout and knockdown cells in vitro where it can take at least 72 h following CreER activation before talin1 protein levels are reduced sufficiently to elicit changes in cell morphology (Fig. S1B and Kopp et al., 2010). However trypsinisation and replating of the cells, which disrupts adhesion complexes, facilitates the loss of talin1 as well as the appearance of defects in cell spreading (Fig. S1C, arrows).

The function of talin has been studied extensively in cells in culture, and it has become apparent that while talin1 is not required for initial cell adhesion and cell spreading, it is required for cells to maintain their spread morphology and for cell migration (Kopp et al., 2010; Zhang et al., 2008). Here we describe for the first time how talin null cells behave in vivo. Visualisation of endothelial cells in *Tln1*^fl/fl^;*Tie2-Cre* embryos by confocal microscopy and TEM show that these endothelial cells are rounded and unable to flatten and spread. The phenotype of the talin1 null cells in vivo is very similar to that of cultured endothelial cells (HUVEC) where knockdown of talin1 prevents focal adhesion formation, and as a result, the cells are unable to maintain a spread morphology. In addition, talin1 knockdown in HUVEC also leads to defects in cell migration (Kopp et al., 2010). Based on these observations, we conclude that in the absence of talin1, endothelial cells in vivo cannot form the cell–matrix junctions required to adopt the flattened morphology found in vessels. Additionally the morphology of *Tln1*^fl/fl^;*Tie2-Cre* endothelial cells in electron micrographs (Fig. 5B) bears considerable similarity to those in a recent report in which it was shown that β1-integrin is required to establish endothelial cell polarity and lumen formation (Zovein et al., 2010). This raises the possibility that talin1 (via β1-integrins) may play a role in these processes as well.

Why endothelial cells, like cells of the haematopoetic lineage such as platelets and dendritic cells express only talin1 (Lämmermann et al., 2008; Petrich et al., 2007) is unclear. Endothelial cells and haematopoetic cells arise from the same progenitor cells, the haemangioblast, during embryonic development (Pardanoud et al., 1989). EST data suggest that both talin isoforms are widely expressed prior to this suggesting that talin2 is specifically turned off in cells of this lineage. The *Tln2* promoter lies within a CpG island (Debrand et al., 2009) raising the possibility that it could be transcriptionally repressed by methylation. Consistent with this idea, down-regulation of talin1 in endothelial cells in vitro does not lead to upregulation of talin2 (unlike in mouse embryo fibroblasts) (Kopp et al., 2010), and thus talin1-deficient endothelial cells show defects in cell spreading, migration and FA assembly. As such endothelial cells provide a system well-suited to conduct detailed structure/function analysis of talin1 in vitro (Kopp et al., 2010) and ultimately, in vivo.

The following are the supplementary materials related to this article.

Supplemental Video 1. 3D view of control vessels. Movie of a rotating 3D-projection of the same region of the *Tln1*^fl/+^;*Tie2-Cre* PECAM1 stained embryo head vessels shown in Fig 5A, b imaged using Leica SP5 confocal microscope and processed using Imaris software.

Supplemental Video 2. 3D view of talin1 mutant vessels. Movie of a rotating 3D-projection of the same region of the *Tln1*^fl/fl^;*Tie2-Cre* PECAM1 stained embryo head vessels shown in Fig 5A, e imaged using Leica SP5 confocal microscope and processed using Imaris software.
